# Gorham-Stout disease of the malleolus: a rare case report

**DOI:** 10.1186/s12891-019-3027-9

**Published:** 2019-12-31

**Authors:** Chuanxi Zheng, Fan Tang, Li Min, Yong Zhou, Yi Luo, Chongqi Tu, Shiquan Zhang

**Affiliations:** 1 Department of Orthopedics, West China Hospital, Sichuan University, Guoxue Xiang No. 37, Chengdu, Sichuan 610041 People’s Republic of China; 2 Department of Joint and Musculoskeletal Tumor, The First Affiliated Hospital of Shenzhen University, Sungang west road No. 3002, Shenzhen, Guangdong 518000 People’s Republic of China

**Keywords:** Gorham-Stout disease, Osteolysis, Bisphosphonate, Biological reconstruction

## Abstract

**Background:**

Gorham-Stout disease, also known as vanishing bone disease, idiopathic massive osteolysis, is a rare entity of unknown etiopathology. This disease is characterized by destruction of osseous matrix and proliferation of lymphatic vascular structures and associated with massive regional osteolysis. It has a variable clinical presentation and is commonly considered as a benign disease with a progressive tendency and an unpredictable prognosis. The diagnosis is made by exclusion and based on combination with histological, radiological, and clinical features. Despite that several therapeutic options have shown certain efficacy, the effective treatment still remains controversial and there is no standard treatment to be recommended.

**Case presentation:**

A previously healthy 40-year-old man presented with right lateral malleolus pain after an ankle sprain and was referred to our hospital. The radiographs indicated rapid massive bone destruction in the distal right lateral malleolus with an unclear margin. Based on the combination with histological, radiological, and clinical features, the diagnosis of Gorham-Stout disease was made. Considering that the residual function of malleolus had to be protected, prior bisphosphonate was used to control the progression of lesion, followed by surgical resection and biological reconstruction with autologous fibular bone grafting. The patient was followed up 8 years after surgery, he presented without progression and recurrence.

**Conclusions:**

We depict a case of Gorham-Stout disease at the right lateral malleolus and was successfully controlled by medication and surgical intervention. Based on the prior effective medical treatment, resection with biological reconstruction is a useful approach to treat Graham-Stout disease in bone.

## Background

Gorham-Stout disease (GSD) is an extremely rare disease characterized by spontaneous osseous matrix destruction and proliferation of lymphatic vascular structures, associated with massive regional osteolysis [1–3]. Presently, only approximately 300 cases of GSD have been reported [4]. Despite the extensive investigation of the pathogenetic mechanisms, its etiology still remains obscure. Unlike other types of idiopathic osteolysis, GSD may develop from 1 month to 71 years old [5–7], with most cases presenting in the second to third decades of life, without gender or race predilection. A previous history of trauma has been described as a trigger of osteolysis and blamed for the etiology of GSD [8, 9].

This disease involves a large spectrum of bones, and the major affected anatomical sites are the maxillofacial, humerus, scapula, ribs, pelvis and femur [5, 10, 11]. The clinical presentation of GSD depends on the anatomical distribution and the span before diagnosis. Common manifestations are pain, swelling and progressive function impairment of the affected region in limb. In cases of ribs, sternum, or thoracic spine involvement, potentially lethal respiratory insufficiency can be caused by instability of the thoracic cage and chylothorax. Paraplegia and fatal outcomes may occur when cervical vertebrae is affected, with an average mortality of 13% [7]. In some cases, the disease manifests acutely, with incapacitating pain, while some other cases present an asymptomatic course, with the diagnosis made after a spontaneous or traumatic fracture.

Radiographs, computed tomography (CT), magnetic resonance imaging (MRI), bone scintigraphy and biopsy are essential items for the diagnosis of GSD. Despite there is no official standardization for the diagnosis of GSD, according to Heffez et al., there are eight recommend diagnostic criteria of GSD (Table 1). The definitive diagnosis is made after excluding a diagnosis of potential infectious, inflammatory, endocrine and malignant, especially, eosinophilic granuloma in children or adolescents, multiple myeloma (MM) in adults. Current treatment modalities include medical treatment with bisphosphonates, vitamin D, and biological drugs; surgical resection followed by reconstruction, radiotherapy, or combination treatments [12–16]. However, there are no standard therapeutic approaches for this disease. In this article, we present a clinical case of GSD involving the malleolus, which is successfully controlled by prior bisphosphonates followed by surgical resection and biological reconstruction.

**Table 1:** Criteria proposed by Heffez et al. for the diagnosis of Gorham-Stout disease

	Criteria	Clinical case
1	Positive histological findings for proliferation and angiomatous dysplasia	✓
2	Absence of osteoblastic reaction and/or dystrophic calcifications	✓
3	Evidence of local bone progressive resorption	✓
4	Exclusion of cellular atypia	✓
5	Non-ulcerative lesion	NA
6	Absence of visceral involvement	✓
7	Osteolytic radiographic pattern	✓
8	Negative hereditary, metabolic, neoplastic, immunologic, and infectious etiology	✓

*NA* Not available

## Case presentation

**Fig. 1:**
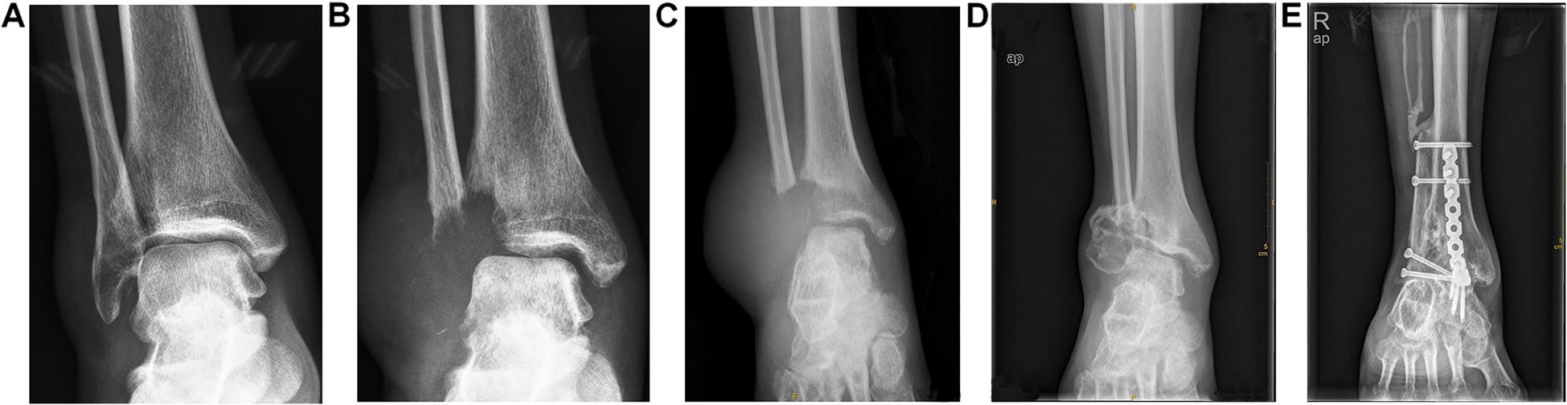
Radiographs images of the right lateral malleolus over the course of treatment. **a** Anteroposterior radiograph of the patient at the first visit after trauma; **b** two months after the trauma, radiograph shows massive osteolysis and obvious soft-tissue edema at right distal tibiofibular; **c** one month after medical treatment, radiograph shows arrest of the osteolysis process; **d** seven months after medical treatment, radiograph shows shrinkage of the lesion and ossifications and sclerosis; **e** eight years after reconstruction surgery, radiograph shows rigid internal fixation with primary union without disease progression

**Fig. 2:**
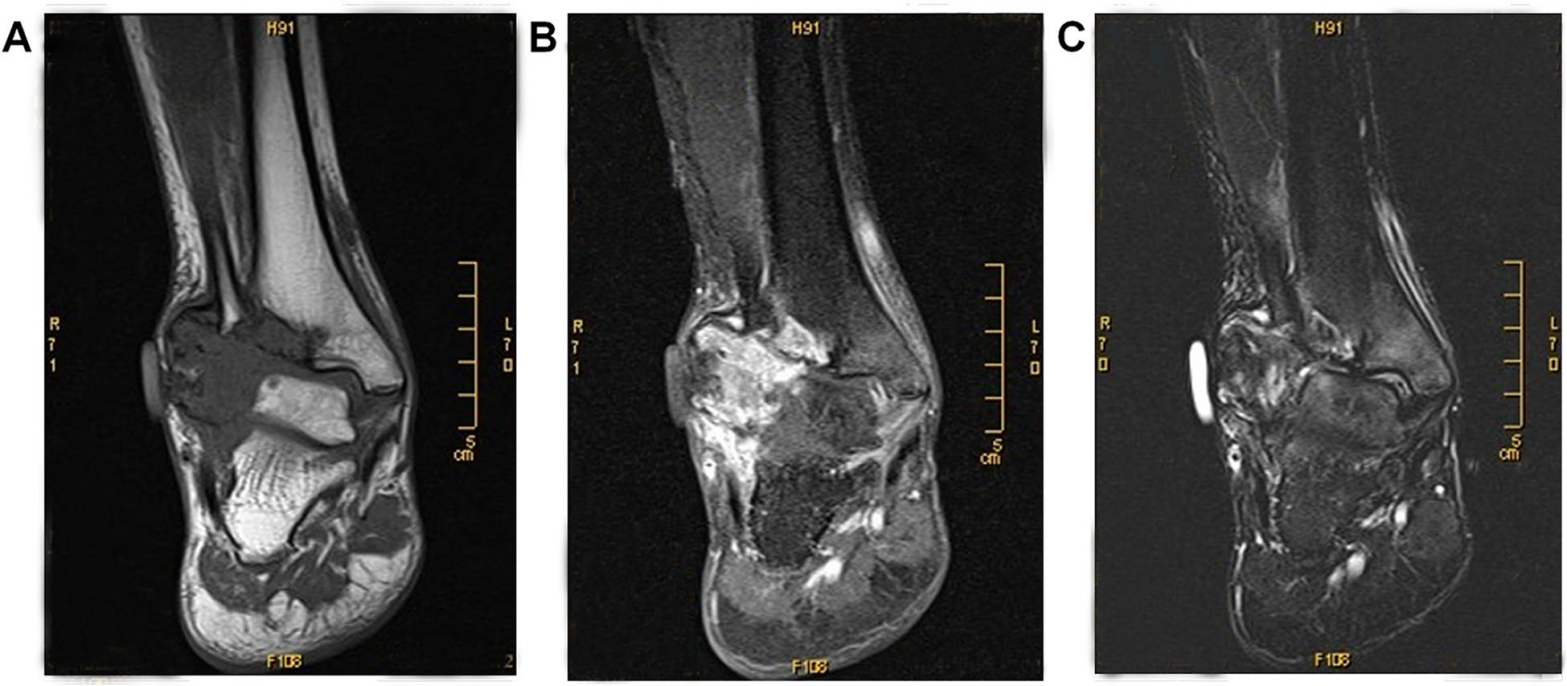
MRI images of massive osteolysis lesion in the right lateral malleolus. **a** Coronal T1-weighted image shows extensive hypointensity lytic lesions involving the right distal of fibula and partial lateral tibia; **b** Coronal fat-suppressed T2-weighted images show extensive hyperintensity in lytic lesions; **c** Coronal T1-weighted images with contrast shows heterogeneous contrast enhancement of the lesion

A previously healthy 40-year-old man presented with right lateral malleolus pain and swelling after an ankle sprain 2 months ago. He visited a primary clinic, and the radiographs showed an avulsion fracture at the right lateral malleolus (Fig. 1a) and the affected limb was immobilized with a short leg cast. Because the symptoms had progressively worsened, he was referred to our hospital for further treatment. At the first presentation, swelling and tenderness were observed, the skin temperature arisen was recognized in the right lateral malleolus and the right ankle range of motion was limited. The radiographs indicated massive bone destruction in the right distal fibula and partial lateral distal tibial cortex with an unclear margin (Fig. 1b). MRI showed a lesion with low signal on T1-weighted image, high signal on T2-weighted image, and peripheral enhancement with gadolinium. These radiological findings corresponded to the malignant bone tumor with an aggressive lytic lesion and associated fluid accumulation (Fig. 2). The patient had no special family history that could guide the diagnosis and no other lesions in the remaining bone. The plain chest radiograph was normal. Calcium, phosphorus and alkaline phosphatase, parathyroid hormone (PTH) level, liver, and kidney function were all within normal limits.

**Fig. 3:**
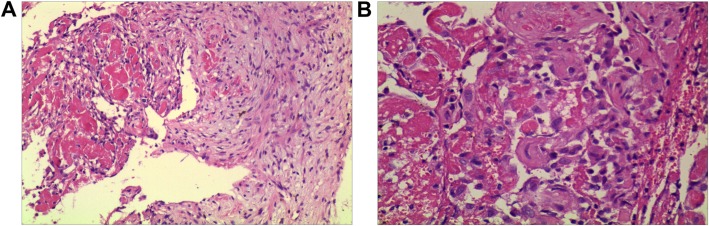
Pathological images of massive osteolysis lesion in the right lateral malleolus. **a** Bone tissues were invaded by areatus hyperplastic blood vessels. (HE × 100); **b** Proliferative capillaries and vascular endothelium formed single-cell lumens and osteoclastic cells were found around the destructed bone trabecula (HE × 200)

**Fig. 4:**
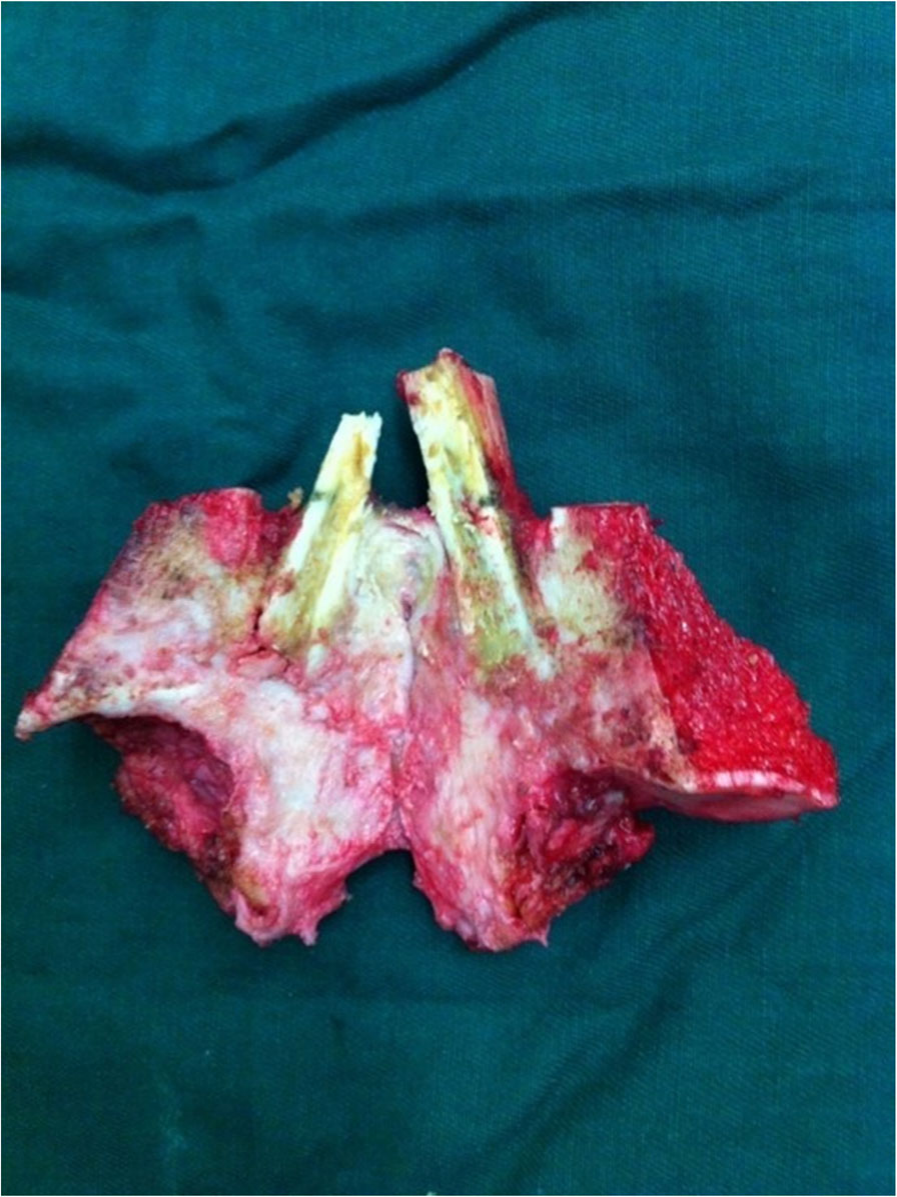
Intraoperative findings after resection of the lesion

A biopsy was performed in the lateral distal fibula lesion. Histological examination revealed that the lesion presented with multiple thin-walled capillary-like vascular channels without cellular atypia, focal aggregations of osteoclastic multinucleated cells associated with giant cells, and intermixed with fibrous connective tissue, mild lymphocytes infiltration. Meanwhile, Osteoblast-osteoclast activity was observed (Fig. 3). According to the diagnostic criteria proposed by Heffez et al., the patient fulfillment of seven out of the eight criteria (Table 1). Then, a diagnosis of GSD was made based on combination with histological, radiological, and clinical features. Subsequently, the patient was treated with intravenous drip bisphosphonate (4 mg/month), calcium(500 mg) and vitamin D (400 UI) once a day. The significant remission of symptoms was achieved after one-month treatment and radiographs showed that bone destruction was controlled (Fig. 1c). After 7 months of medical treatment, regression of lytic lesions and reossificiation were observed in radiographs (Fig. 1d). The radiographs demonstrated shrinkage of the lesion and the border of lesion becomes well-defined with sclerotic rim. Nevertheless, a limited range of motion of right malleolus still remained and the patient complained of intense pain when he walked with full weight-bearing. In order to restore the stability of malleolus, the treatment was proceeded with en-bloc resection lesion followed by reconstructive surgery. An ankle arthrodesis surgery was performed with contralateral autologous fibular bone grafting, internal fixation with bridge plate and screws (Fig. 4). At the last follow-up 8 years after surgery, the patient complained of no pain in the right ankle when he went up or down stairs or walked long distances. Radiographs show that rigid internal fixation with primary union and no local recurrence (Fig. 1)e.

## Discussion and conclusions

Gorham-Stout disease is a rare entity characterized by spontaneous progressive resorption of bone matrix, which is replaced by proliferative vascular and lymphatic canals and subsequently replaced by fibrous tissue.

The tibial and fibular involvement as presented in our case is an uncommon location for GSD. Meanwhile, this case also represents one of the few GSD involving the joints (less than 5 cases had been described in the literature, but malleolus was not referred). The pathogenesis of this disease has not been fully elucidated. Although some studies have described several patients with a history of preceding trauma like our cases, the association of this disease with antecedent trauma has not been clarified [5, 9]. The hypothesis is that GSD might be triggered by a trauma, resulting in an imbalance between the activities of osteoblasts and osteoclasts to induce the isolated but progressive bone resorption [17–19].

Since GSD is a diagnosis by exclusion, the final diagnosis is established based on clinical features, radiological and histological findings. To reach a proper diagnosis clinician need to exclude other differential diagnosis, such as potentially infectious, inflammatory, metabolic or endocrine disease and malignant tumors. Additionally, GSD must also be distinguished from other idiopathic osteolysis classification because it is nonhereditary without concomitant nephropathy [20]. Hyperparathyroidism is the common endocrine disorders with potential complications on the musculoskeletal system, eventually leading to pathological fractures. Therefore, PTH level, calcium and phosphorus values should be included in laboratory tests for differential diagnostics of GSD. Some neoplastic processes which can cause the osteolysis should also be noticed, such as angiomas, aneurysmal bone cyst, eosinophilic granuloma in children.

In the radiographs, four distinct stages of GSD can be recognized [21]. The first stage shows patchy osteopenia in the intramedullary or subcortical regions resembling osteoporosis. At the second stage, progressive osteopenia and cortical erosion without sclerosis. The third stage is characterized by adjacent soft tissue involving. At the final stage, the affected bone is complete resorption and filled with fibrous tissue. The bone window setting and 3-dimensional CT images are useful to depict the extent of bone destruction. Although, MRI can distinctly depict the extent of lesion but is nonspecific. MRI shows hypointensity and hyperintensity on T1 and T2-weighted images, with heterogeneous peripheral gadolinium enhancement. These findings and soft-tissue involvement usually mislead to a diagnosis of malignant tumor, especially multiple sites and pelvic involvement, such as Ewing’s sarcoma, metastasis, osteosarcoma, and multiple myeloma. Bone scintigraphy and whole-body positron emission tomography (PET) imaging can reflect the bone metabolic activity of regional lesion which are extremely useful to rule out the neoplastic or infective pathology [22–24]. Recently, Fluorine-18-sodium fluoride (^18^F-NaF) PET/CT has been proposed as a more bone-specific agent than Fluorine-18-Fluoro-D-glucose (^18^F-FDG) PET/CT and may be a useful approach for GSD diagnosis as well as assessment of disease activity and treatment response [25].

The patient in this study was followed up 8 years without progression. But the clinical course of GSD is unpredictable. It varies from being self-limiting, with mild manifestations, to a fatal outcome [10]. Despite GSD is a benign disease, it can cause fatal outcomes when the crucial anatomical location was affected, such as cervical spine, mediastinum, ribs.

There are no definitive therapy recommendations for GSD. Medical therapy including bisphosphonate, vitamin D, interferon-alpha-2b, calcium, the anti-VEGF-A antibody bevacizumab, low molecular weight heparin, steroids can stabilize the bone destruction and halt the progression of the disease [26–30]. According to the literature, bisphosphonate has been shown to be more effective in the treatment of GSD compared with other regimens [26, 31, 32]. It increases the apoptosis of osteoclasts, diminishes the osteoclast precursor cells amount and reduces the activity of osteoclasts with no action on bone formation. However, there is no consensus about adequate doses, frequency and duration. Monthly i.v. injection is mostly preferred but the duration of treatment differs in the literature [9, 32–34].

Although radiotherapy has been reported to be successful in some radiosensitive cases, the results are controversial and there is no optimal radiotherapy dose for GSD [35, 36]. German Cooperative Group on Radiotherapy for Benign Diseases concluded that radiation therapy can effectively prevent progression of GSD in 80% of case, and advocated dose range of 30 to 45 Gy at 1.8 Gy to 2 Gy per fraction [37]. Of note, radiation could provoke serious side effects, like secondary malignancy and growth restriction in children and adolescents who received high-dose therapy [11, 38].

Surgical treatments are recommended for pathological fractures or reconstruction of massively destroyed bones. Surgery for this disease includes resection alone, resection with endo-prosthetic reconstruction, and resection with biological reconstruction (autogenous bone grafting or allogenous bone graft). Resection alone may be suitable for some dispensable anatomical architectures, such as iliac bone, clavicle, proximal fibula, scapula. While for weight-bearing bone, such as femur, tibia, humerus, reconstruction is mandatory. In our case, the lateral malleolus (distal fibula and partial lateral distal tibia cortex) was involved, talus and medial malleolus were intact, but stability and range of motion of right malleolus were impaired. MRI shows the lesions with an obscure border and regional soft tissue edema which are not suitable for resection. The prior bisphosphonate treatment was applied to control the progression of lesion and regional edema. After 7 months of medical treatment, reossificiation makes the extent of lesion become distinct, therefore resection surgical treatment can be considered. We decided to perform the ankle arthrodesis with autologous fibular bone grafting matching the resected bone.

Ellati R et al. conducted a literature review including 64 cases of GSD and there were 29 cases that received surgical treatment. The follow up results showed that reconstruction with prosthesis cases were effectively controlled without recurrence, but all reconstruction with bone graft cases were progressive [13]. Consistent with results from previous reports, reconstruction with bone graft increase the risk of recurrent bony resorption and shows a low success rate in controlling the progression of the disease [5, 39]. However, an eight-year follow up of our patient, the disease was controlled, and no recurrent resorption of bone was noted. We suppose the probable reason for routine biological reconstruction failure is a high risk of residual lesion during operation, and it easily causes recurrent osteolysis. It is important that the timing of resection with reconstruction should depend on the degree of controlling this disease. The reoccurrence of ossifications and bone regrowth indicate the arrest of osteolysis, and thereafter a surgical therapy for GSD can be considered. Ossifications and sclerosis make the extent of lesion distinct that simplifies the intraoperative manipulation, prevent unintended burst of the tumor and avoid lesion residual. Meanwhile, regression of soft tissue swelling can help to reduce the incidence of wound complication. With regard to some special anatomical location (such as malleolus, radiocarpal joint, shaft of radius and ulna), biological reconstruction will be more suitable than endo-prosthetic reconstruction.

In conclusion, although the previous studies demonstrated that biological reconstruction alone is not effective to control the disease, combining medication with biological reconstruction may constitute an effective approach of treatment. This novel combination was not previously reported in literature for the treatment of GSD, and show a result of well-controlling the disease and no progression at eight-year follow up.

## Data Availability

All data used or analyzed during this study are included in this published article.

## Abbreviations

^18^F-FDG: 18-Fluoro-D-glucose
^18^F-NaF: Fluorine-18-sodium fluoride
CT: Computed tomography
GSD: Gorham-Stout disease
HE: Hematoxylin and eosin
MM: Multiple myeloma
MRI: Magnetic resonance imaging
PET: Positron emission tomography
PTH: parathyroid hormone
T1WI: T1-weighted imaging
T2WI FSE FS-DIXON: T2-weighted imaging fast spin echo frequency selective-Dixon
T2WI: T2-weighted imaging
